# Effects of Mindfulness-Based Cognitive Therapy on Stigma in Female Patients With Schizophrenia

**DOI:** 10.3389/fpsyt.2021.694575

**Published:** 2021-07-23

**Authors:** Qiubi Tang, Shuixian Yang, Cuixia Liu, Liyan Li, Xiaodong Chen, Fengchun Wu, Xingbing Huang

**Affiliations:** The Affiliated Brain Hospital of Guangzhou Medical University, Guangzhou, China

**Keywords:** mindfulness, perceived stigma, coping orientation, cognitive situation, attitude toward mental illness

## Abstract

Mindfulness-based cognitive therapy (MBCT) has been increasingly recognized as effective in different mental illnesses, but these effects are limited in schizophrenia. For patients with schizophrenia, stigma is one of the most negative factors that affects treatment, rehabilitation and social function. This research aimed to determine the effects of MBCT on stigma in patients with schizophrenia. In total, 62 inpatients with schizophrenia were recruited and randomly assigned to the experimental group or control group. The experimental group received an 8-week MBCT intervention, and the control group were treated as usual. Link's Stigma Scales (with three subscales, including perceived devaluation-discrimination (PDD), stigma-coping orientation, and stigma-related feeling), Five Facet Mindfulness Questionnaire (FFMQ), and Insight and Treatment Attitudes Questionnaire (ITAQ) were used to collect data before and after intervention. After intervention, the post-test score of PDD, stigma-coping orientation, FFMQ, and ITAQ were significantly different between the experimental group and the control group. In the experimental group, the PDD and stigma-coping orientation scores significantly decreased, and FFMQ and ITAQ scores increased remarkably (*P* < 0.05). In addition, correlation analysis revealed a significant negative correlation between mindfulness and stigma. MBCT was effective in reducing stigma in patients with schizophrenia, which mainly manifested as changes in the patients' perception of stigma as well as the withdrawal and avoidance caused by schizophrenia. Enhancing mindfulness will help reduce the stigma level. MBCT is worthy of promotion and application in patients with schizophrenia.

## Introduction

Schizophrenia is a serious chronic disease that begins at an early age and negatively affects emotions, thoughts, and behaviors, causing individuals to withdraw from their relationships (1, 2). Schizophrenia has been identified as a priority disorder given its association with high levels of disability and premature mortality, human rights violations and loss of productivity (3). To date, the treatment and rehabilitation of patients with schizophrenia remains a global problem (4). Although antipsychotic drugs can control the psychotic symptoms of illness to a certain extent, the recurrence rate of the disease is very high once a patient stops taking his/her drugs. Most patients need to take medicine for a long time to keep their condition stable (5). Eventually, some patients with schizophrenia still continue to experience disabling residual symptoms and impaired functioning.

Previous studies concluded that stigma is an important factor affecting symptom management, insight into the illness, adherence to treatment and overall function in participants with schizophrenia (6, 7). Patients with schizophrenia experience high levels of stigma, which can be internalized (8). Internalized stigma, or self-stigma, has been defined as “shame, evaluative thoughts, and fear of enacted stigma that results from individuals' identification with a stigmatized group” (9, 10). Stigma is a multifaceted construct that involves feelings, attitudes and behaviors. Stigma comprises three main components: negative stereotypes, prejudice, and discrimination (11).

Stigma is one of the most negative factors that affect the treatment, rehabilitation and social function of patients with schizophrenia (12). Under the influence of the stigma, patients will refuse to accept assistance and systematic specialist treatment. Thus, patients miss the best opportunity for treatment. Some patients are cured but experience frustrations with employment and interpersonal relationships after discharge, which eventually leads to the deterioration of mental illness (13). Thus, it is necessary to perform a scientific and systematic psychological intervention project to reduce the stigma of patients with schizophrenia. However, to date, reports on psychological interventions to reduce the stigma of patients with schizophrenia in China are limited. The nursing interventions performed in the clinic primarily involve the implementation of the doctor's advice and ordinary basic nursing, and the effects are not ideal. Therefore, it is of great significance to explore the effect of mindfulness-based cognitive therapy (MBCT) on stigma in patients with schizophrenia.

Patients with schizophrenia who are more mindful experience self-stigmatizing thoughts less (14). Moreover, some scholars stated that mindfulness-based interventions may be uniquely suited to alleviating distress related to symptoms and internalized stigma in patients with schizophrenia (15). These features may be closely related to the characteristics of mindfulness. Mindfulness, which originated from meditation in Oriental Buddhism, emphasizes the awareness of the present consciously and without evaluation (16). The core methods of mindfulness practice include focusing on the present and not judging what appears in the mind. Mindfulness therapy is a series of psychotherapy sessions based on “mindfulness.” Currently, the most commonly used treatment methods related to mindfulness include mindfulness-based stress reduction (MBSR), MBCT and dialectical behavior therapy (DBT). Inspired by the MBSR program and enriched with elements from cognitive and behavioral therapy (CBT), MBCT was designed by Teasdale, Segal and Williams in the 1990s to treat depression and above all to prevent depressive relapse (17). Although MBCT was initially used to treat depression, an increasing number of scholars were interested in applying this technique to the rehabilitation and treatment of patients with schizophrenia in recent years (18). Therefore, it is quite necessary and important to explore the effects of MBCT on stigma of patients with schizophrenia.

## Methods

### Design and Setting

In order to determine the effects of MBCT on stigma in patients with schizophrenia, we developed this randomized controlled trial that included the interventional and control groups that based on the scores measured before and after intervention. The participants were recruited from the Affiliated Brain Hospital of Guangzhou Medical University, which was the first psychiatric hospital in China with approximately 2000 beds. Based on the survey that a total of 210 patients were investigated with the Brief Psychiatric Rating Scale (BPRS), participants were enrolled by the psychiatrists.

Research participants met the following inclusion criteria: patient was diagnosed with schizophrenia according to the Structured Clinical Interview for International Classification of Diseases-Tenth Revision (ICD-10) diagnosis criteria; patient was stable or in recovery (score on the BPRS <35), but still need to take medicine under the guidance of psychiatrists; patient has not participated in a mindfulness-based intervention program before; age ≥ 18 years; inpatient; and patient is able to communicate in Chinese. Exclusion criteria included the following: participants with organic brain syndrome and patients with hearing, smell and vision problems that prevent the patient from completing the program.

Before the intervention, we used the power analysis to determine the appropriate study sample. The sample size was found to have a level of significance of 0.05 and the effect size of the population representation of 0.92, the sample size was determined to be 54 (27 experimental and 27 control). Thus, the research was conducted with a total of 62 participants who met the inclusion criteria. Participants were randomly divided into an experimental group and a control group using a computer-based random number allocation method, yielding 31 cases in the experimental group and 31 cases in the control group. To ensure homogeneity between groups in term of characteristics that may have affected the research results, matching and randomized methods were used together. We analyzed the differences in marriage, age, and other factors between the two groups, and the research requirements were met.

### Measures

#### Personal Information

This information included age, religion, marital status, duration of disease, whether this is the first episode of schizophrenia, number of hospitalizations in the past 2 years, and length of hospital stay.

#### Five Facet Mindfulness Questionnaire

The FFMQ is a 39-item comprehensive measure of mindfulness level. It assesses five factors of mindfulness, including observing, describing, acting with awareness, non-judgment of inner experience, and non-reactivity to inner experience. Each item score ranges from 1 (never or very rarely true) to 5 (very often or always true). The FFMQ has good construct validity and reliability, and the five sub-facets evidenced good internal consistency in the current sample (Cronbach's alphas range from 0.79 to 0.86) (19).

#### Link's Stigma Scales

This scale consists of three subscales: perceived devaluation-discrimination (PDD), stigma-coping orientation, and stigma-related feeling. Each item score ranges from 1 (strongly disagree) to 4 (strongly agree); higher scores indicate greater stigma perception. The stigma-coping orientation scale included secrecy, withdrawal, challenging, distancing, and educating (20). Stigma-related feelings evaluate the individual's perception of being misunderstood, different from others and ashamed about the illness. The Cronbach's alpha of PDD, stigma-related feeling, and stigma coping scales were 0.89, 0.73, and 0.88, respectively (20).

#### Insight and Treatment Attitudes Questionnaire

The ITAQ is a nurse-administered rating scale proposed by Professor McEvoy in 1989 to evaluate the level of participant insight and treatment attitudes (21). The questionnaire assesses 11 items rated on a three-point Likert scale (0, no insight; 1, partial insight; and 2, good insight). Taking the total score of the scale as the observation index, a higher score indicates better insight and treatment attitude. The Chinese version indicated satisfactory internal consistency (Cronbach's a = 0.82), interrater reliability (ICC = 0.82) and correlations with symptom severity and psychopathology (Pearson's *r* = 0.56 and 0.60, respectively, *P* = 0.001) (22).

The pre and post intervention evaluations were conducted by two regular researchers who did not know whether the participants belonged to the experimental group or the control group. Before the study, they need to receive unified training on the evaluation methods and the understanding of questionnaire.

### Interventions

This study is a randomized, controlled, parallel, partially masked (The rater did not know whether the participants belonged to the experimental group or the control group.), 8-week interventional clinical trial. The 8 weeks of MBCT is mainly provided to improve participants' self-awareness and teach participants how to accept the present, live in the present, live with difficulties and take good care of themselves. The participants in the experimental group were randomly divided into three independent subgroups, including 10 participants in subgroup 1, 11 in subgroup 2, and 10 in subgroup 3. The times at which interventions were provided for each subgroup are presented in Table 1. This three subgroups were led by four mindfulness therapists with MBCT training certificates. The arrangement of weekly intervention content for the experimental group is presented in Table 2. Concomitant treatments among the experimental group and the control group mainly includes drug therapy and the routine rehabilitation nursing activities, such as watching TV and reading books. And there was no difference between the two groups.

**Table 1:** Time of interventions for each group.

**Time**	**Monday (11:30–13:30)**	**Wednesday (11:30–13:30)**	**Friday (11:30–13:30)**
Group	Subgroup 1	Subgroup 2	Subgroup 3

**Table 2:** Arrangement of weekly intervention content.

**Time**	**Theme**	**Purpose**	**Content**
First week	Awareness and automatic navigation	Let the patient realize the automatic navigation mode and start to perceive the body	Mindfulness eating; body scan; patients were introduced to the concept of mindfulness and asked to meet the expectations of the MBCT group.
Second week	Live in the mind	Let the patient know that the recurring thoughts tend to control our reactions to daily things	Awareness of thoughts and emotions; body scan; mindfulness breathing.
Third week	Gather debunching minds	Let the patient be familiar with the operation mode of the mind, return to the anchor cable in the moment, and improve awareness of the body	Stretching in a lying position, visual/auditory awareness, detecting breath and body in a sitting position, responding to the feeling of body pain
Fourth week	Identify boredom	Encourage patients not to pursue or avoid some things painstakingly.	Mindfulness walking and long-term meditation; awareness of breath, body feeling, voice and thought; non-selective awareness.
Fifth week	Accept/Let it be	Allow difficulties to exist and live with them	Awareness of breathing, body feeling and difficulties in the exercise process.
Sixth week	Thoughts are not facts	Let the patient realize that thoughts are just thoughts. Emotions and the corresponding thoughts will affect our responding ability.	Awareness of breathing, body feeling and unpleasant sounds, thoughts and emotions
Seventh week	How to take care of yourself	Let the patient understand the nourishing and consuming events in their life and take action to take care of themself.	Awareness of breathing, body feeling and unpleasant sounds, thoughts and emotions.
Eighth week	Keep learning	Regular mindfulness practice helps to maintain the balance of life and realize the importance of persisting in learning.	Body scan; program summary.

### Statistical Analysis

Descriptive analysis was performed to calculate the scores of each of the measurement scales. Comparisons of the intervention and control groups at baseline for the demographic data was performed using one-way ANOVA. Dependent-sample *t*-tests were performed to compare the pre/post intervention mean scores of the scale between the intervention and control group, whereas paired *t*-tests were performed to analyzed the inner group difference of score in each group. Independent-samples *t*-test were used for comparison of mean scores of the scale among participants in the control and experimental groups. Data analyses were performed using the SPSS statistical package, version 21.0 (IBM SPSS Statistics, IBM Corporation, Armonk, NY). All statistical tests were two-sided, and the level of significance was set at *P* < 0.05.

## Result

A total of 62 participants aged between 18 and 66 years were included in the research, including 31 in the experimental group and 31 in the control group. Participants with a duration of illness average (23.38 ± 13.10) years, mainly have the negative symptoms such as apathy, social withdrawal and lack of interest. They were stable and in recovery without the risk of self injury, suicide and impulse. No significant differences in age, religious belief, marriage and course of disease were noted between the two groups. See Table 3 for details. During the implementation of the research, one participant in the experimental group was absent from the course once because she asked to leave the course in the fifth week, so data from this patient were excluded from the final result analysis (except the demographic data). In addition, two participants in the control group dropped out of the study. Thus, at the end of the experiment, there were 30 participants in the experimental group and 29 in the control group.

**Table 3:** Demographic data of the control group and experimental group.

**Variable**	**Overall**	**experimental group**	**Control group**	***t/z***	***P***
Age (years, $\bar{x}$ ± s)	47.65 ± 12.56	47.16 ± 11.99	48.13 ± 13.29	0.301	0.764
Is there any religious belief?				1.072	0.283
No	53	25	28		
Yes	9	6	3		
Marital status				−0.389	0.697
Unmarried	40	19	21		
Married	12	7	5		
Divorced	5	3	2		
Widowed	5	2	3		
First-episode schizophrenia				1.026	0.305
Yes	4	1	3		
No	58	30	28		
Disease duration (years, $\bar{x}$ ± s)	23.38 ± 13.10	22.61 ± 13.11	24.16 ± 13.25	0.462	0.645
Number of hospitalizations in the past 2 years	1.15 ± 1.05	1.03 ± 0.19	1.09 ± 0.20	0.599	0.551
Length of hospital stay (months, $\bar{x}$ ± s)	7.27 ± 4.04	7.74 ± 0.79	6.81 ± 0.66	0.911	0.366
Score on the Brief Psychiatric Rating Scale ($\bar{x}$± s)	31.89 ± 2.48	31.90 ± 3.07	31.87 ± 3.14	0.51	0.960

No significant differences in the scores of LINK's stigma scale, FFMQ and ITAQ before the intervention were noted between the experimental group and the control group. After 8 weeks of MBCT intervention, the total score of LINK's stigma scale in the experimental group was significantly lower than that in the control group. With the exception of factors of separation and stigma-related feeling, the scores of the other six factors were significantly different between the experimental group and control group (*P* < 0.05). Similarly, the total FFMQ score in the experimental group was significantly greater than that in the control group and was mainly reflected in three factors of observing, describing, acting with awareness. In terms of ITAQ, the score in the experimental group was also significantly greater than that of the control group (*P* < 0.05). See Table 4.

**Table 4:** Comparison of the two groups before and after intervention.

**Variable**	**Group**	**Pre-intervention ($\bar{x}$ ± s)**	***t/z***	***P***	**Post-intervention ($\bar{x}$ ± s)**	***t/z***	***P***
PDD	IG	31.61, 3.34	−0.111	0.912	28.00, 4.60	−3.653	0.001
	CG	31.71, 3.52			31.28, 1.69		
Stigma-coping orientations	IG	64.00, 6.35	0.384	0.702	56.57, 5.55	−6.206	<0.001
	CG	63.45, 4.79			64.66, 4.37		
Secrecy	IG	21.45, 3.57	0.342	0.733	19.17, 3.79	−2.100	0.040
	CG	21.16, 3.09			21.03, 2.97		
Withdrawal	IG	19.19, 2.29	0.597	0.553	16.97, 2.67	−4.155	<0.001
	CG	18.84, 2.40			19.59, 2.13		
Education	IG	7.58, 1.48	0.777	0.440	6.33, 1.27	−4.531	<0.001
	CG	7.29, 1.47			7.93, 1.44		
Challenge	IG	9.39, 1.45	−0.286	0.776	8.03, 1.45	−3.415	0.001
	CG	9.52, 2.05			9.55, 1.94		
Separation	IG	6.39, 1.05	−0.980	0.331	6.07, 1.17	−1.474	0.146
	CG	6.65, 1.02			6.55, 1.35		
Stigma-related feelings	IG	16.90, 2.70	0.415	0.680	15.93, 3.37	0.346	0.731
	CG	16.65, 2.20			15.66, 2.77		
FFMQ	IG	113.13, 9.91	−0.486	0.628	126.00, 14.33	3.734	<0.001
	CG	114.58, 13.33			112.59, 13.23		
Observe	IG	24.58, 4.96	0.349	0.729	26.97, 4.00	3.028	0.004
	CG	24.20, 3.69			23.66, 4.39		
Describe	IG	23.67, 4.23	0.826	0.412	26.23, 4.00	3.814	<0.001
	CG	22.84, 3.75			22.59, 3.30		
Act with awareness	IG	19.64, 4.99	−0.552	0.583	24.20, 5.54	2.655	0.010
	CG	20.42, 6.01			20.24, 5.91		
Non-judgment	IG	24.71, 3.16	−0.721	0.474	26.23, 3.54	1.236	0.221
	CG	25.39, 4.17			25.03, 3.91		
Non-reactivity	IG	20.52, 3.13	−1.447	0.153	22.37, 2.41	1.738	0.088
	CG	21.74, 3.53			21.07, 3.27		
ITAQ	IG	14.03, 4.28	1.077	0.286	17.27, 2.98	5.202	<0.001
	CG	12.48, 6.76			11.48, 5.28		

*IG represents the experimental group, and CG represents the control group*.

After the intervention, the evaluation indicators of the experimental group and the control group were compared within the group. With the exception of the score of separation and stigma-related feelings in LINK's stigma scale and that of non-judgment of inner experience in FFMQ, all the evaluation indicators were significantly different in the experimental group but not in the control group. See Table 5.

**Table 5:** Within group comparisons for each group before and after the intervention.

**Variable**	**Before or after intervention**	**Control group ($\bar{x}$ ± s)**	***t/z***	***P***	**Experimental group ($\bar{x}$ ± s)**	***t/z***	***P***
PDD	Before	31.71, 3.52	0.601	0.550	31.61, 3.3	3.517	0.001
	After	31.28, 1.69			28.00, 4.60		
Stigma-coping orientation	Before	63.45, 4.80	−1.014	0.315	64.00, 6.3	4.863	<0.001
	After	64.66, 4.37			56.57, 5.55		
Secrecy	Before	21.16, 3.09	0.162	0.872	21.45, 3.5	2.42	0.019
	After	21.03, 2.97			19.17, 3.80		
Withdrawal	Before	18.84, 2.40	−1.274	0.208	19.19, 2.2	3.502	0.001
	After	19.59, 2.13			16.97, 2.67		
Education	Before	7.29, 1.47	−1.708	0.093	7.58, 1.4	3.531	0.001
	After	7.93, 1.44			6.33, 1.27		
Challenge	Before	9.52, 2.05	−0.069	0.945	9.39, 1.4	3.641	0.001
	After	9.55, 1.94			8.03, 1.45		
Separation	Before	6.65, 1.02	0.304	0.762	6.39, 1.0	1.123	0.266
	After	6.55, 1.35			6.07, 1.17		
Stigma-related feelings	Before	15.55, 2.03	−0.171	0.865	16.90, 2.7	1.242	0.219
	After	15.66, 2.77			15.93, 3.37		
FFMQ	Before	114.58, 13.33	0.581	0.563	113.13, 9.9	−4.092	<0.001
	After	112.59, 13.23			126.00, 14.33		
Observe	Before	24.19, 3.69	0.515	0.608	24.58, 4.9	−2.064	0.043
	After	23.66, 4.39			26.97, 4.00		
Describe	Before	22.84, 3.75	0.276	0.784	23.68, 4.2	−2.424	0.018
	After	22.59, 3.30			26.23, 4.00		
Act with awareness	Before	20.42, 6.01	0.116	0.908	19.65, 4.9	−3.378	0.001
	After	20.24, 5.91			24.20, 5.54		
Non-judgment	Before	25.39, 4.17	0.338	0.737	24.71, 3.1	−1.774	0.081
	After	25.03, 3.91			26.23, 3.54		
Non-reactivity	Before	21.74, 3.53	0.764	0.448	20.52, 3.1	−2.58	0.012
	After	21.07, 3.27			22.37, 2.41		
ITAQ	Before	12.48, 6.76	0.636	0.527	14.03, 4.2	−3.411	0.001
	After	11.48, 5.28			17.27, 2.98		

Based on the correlation analysis of the scores of FFMQ, three subscales of stigma and ITAQ, we observed a significant negative correlation between the scores of FFMQ and the Perceived Devaluation Discrimination subscale (*r* = −0.473, *P* < 0.05). A significant positive correlation was noted between the FFMQ and ITAQ scores (*r* = 0.505, *P* < 0.05). In addition, a significant positive correlation was found between the ITAQ and the stigma-coping orientation subscale scores (*r* = −0.323, *P* < 0.05). See Table 6 for details.

**Table 6:** Correlation analysis of mindfulness level with stigma, insight and treatment attitude.

**Variable**	**Observe items**		**Describe items**		**Act with awareness items**		**Non-judgment items**		**Non-reactivity items**		**Total score of FFMQ**		**Total score of ITAQ**	
	***r***	***P***	***r***	***P***	***r***	***P***	***r***	***P***	***r***	***P***	***r***	***P***	***r***	***P***
PDD	−0.260	**0.043**	−0.297	**0.020**	−0.342	**0.007**	−0.351	**0.006**	−0.308	**0.016**	−0.473	<0.001	−0.194	0.134
Stigma-coping orientation	−0.026	0.845	0.079	0.547	−0.275	**0.032**	−0.001	0.993	−0.140	0.280	−0.128	0.325	−0.323	**0.011**
Stigma-related feelings	−0.234	0.069	−0.111	0.395	−0.074	0.569	−0.284	**0.027**	−0.173	0.183	−0.251	0.051	−0.182	0.161
ITAQ	0.478	<0.001	0.437	<0.001	0.061	0.643	0.526	<0.001	0.253	**0.049**	0.505	<0.001		

## Discussion

MBCT can effectively reduce the level of stigma in patients with schizophrenia and improve the level of mindfulness, insight and treatment attitude. As shown in the study, after intervention, significant differences were revealed when the mean scores of stigma, mindfulness level and insight and treatment attitude were compared between the experimental group and the control group. The experimental group's mean stigma scores in the posttest decreased significantly in relation to the control group. FFMQ and ITAQ scores were significantly increased compared with those of the control group. Intragroup comparisons of the mean pre- and post-test scores revealed significant differences between the experimental group's mean stigma score, the FFMQ score and the ITAQ score. The stigma scores in the post-test were significantly decreased compared with pretest scores. FFMQ and ITAQ scores were significantly increase after intervention.

The level of stigma attracts more and more attention, as it has become one of the most important indicators to measure the recovery of schizophrenia patients (23). Previous research showed that when patients with schizophrenia were stable, they could still feel disappointed, isolated and helpless (24). When they are faced with misfortune or setback, the individual's acceptance of their own shortcomings and understanding of their own abilities are defective. In addition, it is difficult for patients to accept their living environment, and their painful emotions and thoughts may continue for a long time. These patients cannot objectively and rationally view their own problems, resulting in excessive bad cognition and a negative or evasive lifestyle (25). All these characteristics further lead to the patients' thoughts of sadness, stigma and even guilt that seriously affect the patients' life satisfaction and social function (10). Similar to many previous studies, the pretest results in this study reveal that schizophrenia patients have a higher level of stigma, which subsequently decreases their levels of life quality, hope, self-esteem, and adherence to treatment (10, 26).

The results of this study show that MBCT can effectively improve the patient's stigma-coping orientation, which is mainly reflected by changes in four factors: secrecy, withdrawal, education and challenge. The secrecy factor reflects a patient's attitude and behavior of concealing his/her condition to avoid rejection by others. After MBCT intervention, patients can accept their own experience of suffering from schizophrenia, treatment and other information related to mental illness. For example, when the patients were interviewed after intervention, they said, now I can tell others my illness and I am willing to make friends on my own initiative. This result may be related to MBCT's advocacy of accepting the present and living in the present. The withdrawal factor reflects whether the patient wants to withdraw or avoids other people and social contact to avoid potential rejection. Patients with schizophrenia often believe that they are sick and different from others. Thus, social avoidance will appear as they worry about rejection by others in the process of socializing. However, patients typically fail to clearly discover their worries, avoidance, or even anxiety, representing the “automatic navigation mode” in their thinking mode. After MBCT intervention, the withdrawal behavior of patients is significantly improved, which may be related to the “existence mode” advocated by MBCT. In the process of intervention, we first let the patients know their own “automatic navigation mode” (for example, in the process of body scanning, attention always runs away, lots of thoughts come and go, and so on) and then teach them to remain aware of their body, emotion and thoughts. In this way, these patients may ultimately identify their own avoidance thoughts. The education factor reflects the attitude and behavior of patients to teach others about mental illness to reduce rejection. The challenge factor reflects the patients' degree of challenge and refutation of the discriminatory behaviors and attitudes of others toward mental illness. The previous study (23) indicated that schizophrenia patients exhibit attitudes and behaviors related to the factors of education and challenges mainly because they excessively or prematurely perceive the discrimination toward themselves. Therefore, MBCT focuses on improving patients' awareness ability by training their awareness of breathing, emotions, thoughts, and body.

In addition to the reduction in the level of PDD and the improvement in the coping orientation, MBCT can also effectively improve the level of mindfulness (according to the FFMQ score), cognitive cognition and attitudes regarding the treatment of mental illness (according to the ITAQ score). As noted in Table 6, the patient's level of mindfulness is negatively related to the PDD. This finding indicates that the higher the patient's level of mindfulness, the lower the level of stigma caused by the disease. The higher the level of mindfulness, the higher the self-awareness and the better the attitude toward treatment. Similarly, the lower the level of PDD, the higher the self-awareness and the better the attitude toward treatment. These three variables are interrelated and influence each other. As noted in the previous study (12, 27), when patients experience a strong sense of stigma, there will be more concealment of their own conditions, isolation from the outside world and other behaviors. Stigma represents a significant obstacle to the long-term treatment of the disease and drug compliance (28). In the process of mindfulness practice, every movement and the inner state of mind of patients should be combined with mindfulness breath, body feeling and awareness. In a clear and quiet state, patients should be guided to observe all types of body feelings subconsciously, directly and objectively. In addition, participants should be in harmony with this experience to aid in the acceptance of external stimulation intuitively.

Finally, we would like to mention that teaching awareness is an important part of MBCT. And it is not easy to assess the level of mindfulness of the patients with schizophrenia. The awareness training need to start with the simplest breathing awareness, and then gradually expand to the awareness of physical feelings, emotions and thoughts. This may help patients to understand the FFMQ more easily. Besides, in the process of assessing the level of mindfulness, we need to explain the content of FFMQ in more detail and list the simple examples in the daily life. For example, whether you are aware of the temperature of the water when you take a bath and the movement of your body when you walk.

## Conclusion

The findings of this research preliminarily support the effects of MBCT on patients with schizophrenia. MBCT can effectively reduce the level of perceived-stigma and improve the coping orientation, the cognitive situation and the attitude of mental illness. A higher the level of mindfulness indicates a better cognitive status and attitude regarding treatment for mental illness. This project is worthy of popularization in patients with schizophrenia.

## Ethical Considerations

Ethical approval to conduct the study was obtained from the Human Research Ethics Committee of Affiliated Brain Hospital of Guangzhou Medical University. We follow the “informed consent” principle that participants are informed about the purpose of our research and their questions were answered. In addition, “self-determination” principle was met by informing participants that they could leave the study and that their information would be kept confidential and would not be used in other places.

## Limitations

The limitations of our research include the small size of the sample, recruitment from a single one location, masking methods and only female patients were included in the study. Thus, it may be difficult to generalize the findings. A large sample or multi-center study should be used in future research. In addition, although no significant difference was noted between the experimental group and the control group at baseline assessment, we were unable to collect the dosage of prescription drugs and relevant information about symptoms or disease onset. These factors should be considered in future research to provide a more rigorous assessment. The main reason why only female patients were included in the study is that the male patients and female patients in our hospital were arranged separately in different wards for treatment. In order to facilitate the study, we only recruited the relatively concentrated female patients for the intervention, without considering gender issues. But it does not mean that MBCT is not suitable for the male patients with schizophrenia or ineffective in male patients.

## Data Availability Statement

The original contributions presented in the study are included in the article/supplementary material, further inquiries can be directed to the corresponding author/s.

## Ethics Statement

The studies involving human participants were reviewed and approved by Affiliated Brain Hospital of Guangzhou Medical University. The patients/participants provided their written informed consent to participate in this study.

## Author Contributions

QT, SY, and XH contributed to the conception and design and wrote the original draft of the manuscript. QT, SY, CL, LL, XC, FW, and XH contributed to manuscript revisions, review and analysis of the literature, and creation of the tables. All authors contributed to the article and approved the submitted version.

## Conflict of Interest

The authors declare that the research was conducted in the absence of any commercial or financial relationships that could be construed as a potential conflict of interest.

## Publisher's Note

All claims expressed in this article are solely those of the authors and do not necessarily represent those of their affiliated organizations, or those of the publisher, the editors and the reviewers. Any product that may be evaluated in this article, or claim that may be made by its manufacturer, is not guaranteed or endorsed by the publisher.
